# Time trends of major headache diagnoses and predictive factors. Data from three Nord-Trøndelag health surveys

**DOI:** 10.1186/s10194-020-01095-5

**Published:** 2020-03-11

**Authors:** Knut Hagen, Lars Jacob Stovner, John-Anker Zwart

**Affiliations:** 1 Department of Neuromedicine and Movement Science, Norwegian University of Science and Technology, Trondheim, Norway; 2 Clinical Research Unit Central Norway, St. Olavs Hospital, Trondheim, Norway; 3 Norwegian Advisory Unit on Headaches, St. Olavs University Hospital, 7489 Trondheim, Norway; 4 Department of Research and Innovation, Division of Clinical Neuroscience, Oslo University Hospital, Oslo, Norway; 5 Institute of Clinical Medicine, University of Oslo, Oslo, Norway

**Keywords:** Migraine, Tension-type headache, Epidemiology, General population, Follow-up

## Abstract

**Aims:**

The primary aim of this study was to investigate time trends of major headache diagnoses using cross-sectional data from two population-based health surveys. In addition, we aimed to perform a longitudinal assessment of baseline characteristics and subsequent risk for having headache at 22-years’ follow-up among those participating in three health surveys.

**Methods:**

Data from the Nord-Trøndelag Health Study (HUNT) performed in 1995–1997 (HUNT2), 2006–2008 (HUNT3) and 2017–2019 (HUNT4) were used. The 1-year prevalence time trends of major headache diagnoses were estimated among 41,460 participants in HUNT4 and among 39,697 participants in HUNT3, two surveys with identical headache questions. 16,118 persons participated in all three surveys, and among these, a Poisson regression was used to evaluate health-related baseline information in HUNT2 and the risk ratios (RRs) with 95% confidence interval (CIs) of consistently reporting headache during follow-up.

**Results:**

Compared with the 1-year prevalence in HUNT3, a higher proportion of participants in HUNT4 had tension-type headache (20.7% vs. 15.9%, *p* < 0.001), whereas a lower 1-year prevalence was found for migraine (11.1% vs. 12.0%, *p* < 0.001) and medication overuse headache (MOH) (0.3% vs. 1.0%, p < 0.001). Participants in the age group 20–39 years at baseline nearly three times increased risk (RR = 2.8, 95% CI 2.5–3.1) of reporting headache in HUNT2, HUNT3 and HUNT4 than persons aged 50 years or more. Female sex, occurrence of chronic musculoskeletal complaints and high score of depression or anxiety at baseline doubled the risk of having headache in all three surveys.

**Conclusions:**

The 1-year prevalence of migraine and MOH was lower in HUNT4 than in HUNT3. Young age, female sex, and occurrence of musculoskeletal complaints and high score of anxiety and/or depression were all associated with substantially increased risk of reporting headache in all three surveys.

## Introduction

A 1-year prevalence of 26% for tension-type headache (TTH) and of 14% for migraine have been estimated globally, higher among female and individuals aged between 20 and 50 years of age [1]. In population-based studies evaluating secular trends in adults the results have been conflicting [2–10]. Increasing incidence of medically recognized migraine was demonstrated in studies from the US performed in the period between 1979 to 1990 [2, 3]. Furthermore, in studies published in the last two decades, increasing migraine prevalence have been found in Hong Kong (from 1.5% to 4.5%), Spain (from 6.5% to 9.7%) and in Norway (from 12.1% to 13.2%). Others have found more stable prevalence rates [6–10]. However, more studies are needed to clarify recent time trends in major headache diagnoses.

Relatively few population-based studies have evaluated the long-term prognosis of headache. In a population-based 12-year follow-up study from Denmark, remission occurred in 40–45% of individuals with previous headache [11]. In a 30 years prospective study from Switzerland, 21% continued to have migraine and 7% TTH for more than half of the follow-up period, whereas most headaches were transient [12]. Furthermore, the 40-years’ follow-up study performed by Bo Bille showed that in a subgroup of 73 children with pronounced migraine, more than half of the migraine group still had migraine attacks around the age of 50 [13].

Identification of prognostic factors of headache is important from both a clinical and a public health perspective [11]. In the Danish follow-up study, high migraine frequency at baseline and young age of onset was associated with poor outcome defined as more than 14 migraine days per year at follow-up [11]. However, the impact of lifestyle factors and comorbid conditions on the risk of having persisting headache after more than 20 years follow-up is unclear.

In the present study, we evaluated time trends of 1-year prevalence of major headache types using cross-sectional data from two health surveys performed with 11-years’ interval, and also evaluated the influence of health-related factors at baseline on the risk of having headache in three surveys with a 22-years’ follow-up.

## Methods

### Study design

This is a population-based study comparing cross-sectional one-year prevalence of major headache types in two surveys performed with a 11 years’ interval. In addition, it also evaluates the impact of baseline factors on the risk of headache after 22-year follow-up in participants in all three surveys.

### The HUNT surveys

The present study included data from three HUNT surveys conducted in Nord-Trøndelag County, Norway, in 1997–1997 (HUNT2) [14], in 2006–2008 (HUNT3) [6], and in 2017–2019 (HUNT4) [15]. The mean follow-up duration between HUNT2 and HUNT4 was 21.7 years. In these three surveys the entire population of the Nord-Trøndelag County aged 20 years of age or more was invited to answer health-related items in two different questionnaires (Q1 and Q2), including questions about headache. The participants were also invited to a clinical examination, involving the measurement of weight, height, and blood pressure [6, 14–16].

### Headache diagnoses

HUNT3 and HUNT4 had 14 identical headache questions. The initial screening question was “Have you suffered from headache during the last 12 months?”, and those who responded “yes” answered 13 additional questions. In the present study slight modifications of the criteria of the International Classification of Headache Disorders, third edition (ICHD 3) [17] was used for diagnosing migraine, probable migraine, tension-type headache (TTH) or medication overuse headache (MOH) (see below) [18]. The diagnoses were mutually exclusive. “Unclassified headache” emerged as an exclusion diagnosis defined by a positive answer to the screening question, but without fulfilling any of the diagnoses mentioned above.

As to the modifications of the criteria, individuals would fulfill the migraine criteria even if the attack lasted less than 4 h, because they were not asked for untreated attacks in the question “How long does the headache usually last?” Migraine with aura was diagnosed in those who fulfilled the migraine criteria and answered “yes” to the question “Are the headaches usually characterized by or accompanied by visual disturbance before or during onset (zigzag lines, flickering/flashing light, blurred vision).” MOH was diagnosed in those with headache ≥15 days/month who reported use of pain killers for headache at least 4 days/week during the last month. In the present study the diagnosis of MOH excluded the diagnoses of chronic TTH, chronic migraine and the chronic part of unclassified headache. This strategy captured all participants with MOH, but it decreased the prevalence of chronic TTH, chronic migraine and unclassified headache.

The headache questionnaire in Q2 of HUNT2 was quite similar to the one used in HUNT3 and HUNT4, including the screening question, except that a question about pain intensity was lacking. Consequently, the definition of migraine used in HUNT2 was stricter regarding migraine criterion C (at least two of three characteristics) than applied in the definition used in HUNT3 and HUNT4 (at least two of four characteristics). Headache sufferers not fulfilling the criteria of migraine were classified as “Unclassified headache”. To fulfil the diagnosis of MOH in HUNT2, individuals should report headache ≥15 days/month and using pain killers daily or nearly daily for at least 3 months during the last year.

Individuals who participated in all three surveys were classified into four groups using categorization published previously [19]. Those who answered “yes” to the screening question in HUNT2, HUNT3, and HUNT4 were classified as “stable headache”. Individuals who reported headache in HUNT2 and/or HUNT3, but not in HUNT4 were grouped as “previous headache”, whereas those who reported headache in HUNT4, but not in HUNT2 were considered as having “new onset headache.” Persons who answered “no” in all three surveys were “headache free controls”.

### Validity of headache diagnosis

We have previously reported the validity of the questionnaire-based headache diagnoses in HUNT2 [20], HUNT3 [21], and HUNT4 [15], respectively. Merged data of HUNT3 (*n* = 293) og HUNT4 (*n* = 232) gave the following results: for headache suffering, the sensitivity was 90% and specificity79% (kappa value 0.60, 95% CI 0.55–0.65); for migraine, the sensitivity was 54% and specificity 95% (kappa values 0.52, 95% CI 0.47–0.57); for migraine with aura, the sensitivity was 39% and specificity 95% (kappa values 0.34, 95% CI 0.30–0.38); for TTH ≥1 days/month, the sensitivity was 97% and specificity was 71% (kappa value 0.38, 95% CI 0.31–0.45), for chronic TTH, the sensitivity was 47% and specificity was 100% (kappa value 0.61, 95% CI 0.53–0.69), and for MOH the sensitivity was 67% and specificity was 100% (kappa value 0.72, 95% CI 0.35–1.00). Correspondingly, in HUNT2, the validity of the questionnaire-based status of being a headache sufferer had a sensitivity of 83% and specificity of 85% (kappa value 0.57, 95% CI 0.41–0.73) [20]. Furthermore, for the restrictive diagnosis of migraine, the sensitivity was 63% and specificity 92% (kappa values 0.57, 95% CI 0.44–0.70), and for unclassified headache, the sensitivity was 61% and specificity 81% (kappa value 0.43, 95% CI 0.29–0.57) [20].

### Study population

**Fig. 1:**
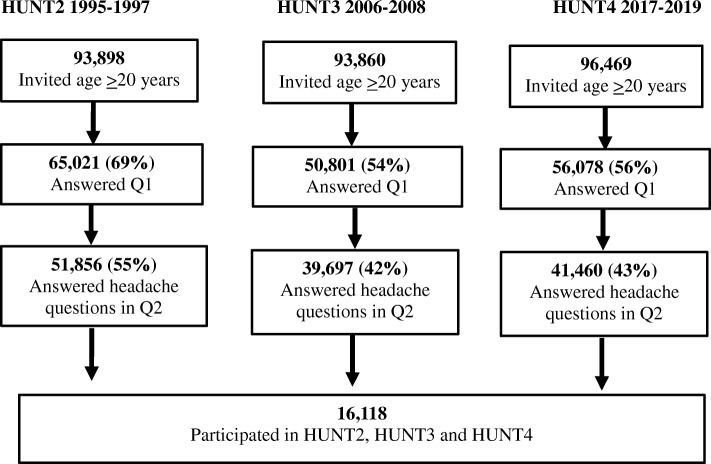
The flow of participants in HUNT2, HUNT3 and HUNT4

The number of invited individuals and responders in HUNT2, HUNT3 and HUNT4 is presented in the flow diagram (Fig. 1). As demonstrated, the number of participants answering the headache question in Q2 was 51,856 (55% of all invited) in HUNT2, 39,697 (42% of all invited) in HUNT3 and 41,460 (43% of all invited) in HUNT4. The proportion of female responders were 54% in HUNT2, 56% in HUNT3 and 57% in HUNT4. A total of 16,116 (17.2% of all invited in HUNT2) answered the headache question in all three surveys (9563 women and 6555 men).

### Baseline factors in HUNT2

HUNT2 included a wide range of health-related information, and several factors have previously been identified to be associated with any headache and migraine [21, 22]. Sex and age (10-years categories); years of education in HUNT2 (≤9, 10–12 and ≥ 13 years); and anxiety and depression as measured by the scores of the total Hospital Anxiety and Depression Scale (HADS) (≤7, 8–10, ≥11). Body mass index (BMI) was calculated based on measurements of weight and height. The BMI were divided into three categories; < 25.0, 25.0–29.9, and ≥ 30.0 kg/m^2^. Regarding smoking the participants were divided into two categories; current daily or previously smoking, and never daily smoking. Regarding physical activity the participants were categorized into three groups based on two questions about light (no sweating or heavy breathing) and hard (sweating and heavy breathing) physical activity; none or less than 1 h of light activity, 1–2 h of light and/or hard activity; and three or more hours of hard activity. As for alcohol use, based on the non-validated question in Q1 “How many times per month do you usually drink alcohol”, the participants were divided into four groups; no use, less than 4 times per month, 4 times/month, or at least 5 times/month.

### Ethics

The present study was approved by the Regional Committee for Ethics in Medical Research (2018/2428 REK midt). The HUNT Study was in addition approved by the Norwegian Data Inspectorate All participants signed a written consent.

#### Statistical analysis

For the various headache types, the crude 1-year prevalence in men and women was estimated in HUNT4. The prevalence of all headache diagnoses was sex- and age-adjusted, using the age distributions of all inhabitants aged ≥20 years invited to HUNT3 as the standard population. Age-and sex adjusted prevalence in the HUNT4 and HUNT3 surveys were compared with the chi-square test. A modified Poisson regression with a robust error variance was used to estimate risk ratios (RRs) of headache status in HUNT4. In accordance with a pre-planned strategy, it was decided to include adjustment for age, sex and duration of education in all analyses. In this way, comparison of RR between different baseline factors was possible. Supplementary analyses were conducted to evaluate the influence of age, dividing the participants into three groups based on age in HUNT2; 20–39 years (0–33 percentile), 40–49 years (34–66 percentile), and 50 years and older (67–100 percentile). Data analyses were performed with the IBM SPSS version 25 (SPSS, Chicago, Illinois, USA).

## Results

One-year prevalence of different types of headache in HUNT4 among women and men are presented in Table 1. Overall, the crude prevalence of headache suffering was 37.2% (95% CI 36.5–37.9), significantly higher among women (45.0%) than men (26.9%) (*p* < 0.001). The crude prevalence of migraine was 11.1% (95% 10.8–11.4), whereas the age-and sex adjusted 1-year prevalence was 11.2% (95% CI 10.9–11.5). The corresponding crude and age/sex-adjusted 1-year prevalence of TTH was 20.7% (95% CI 20.3–21.1). Among those with headache ≥15 days/month, 0.8% (95% CI 0.7–0.9) fulfilled our criteria of chronic migraine, 1.0% (95% CI .0.9–1.1) had chronic TTH and 0.3% (95% CI 0.2–0.3) had MOH. Lifetime prevalence of migraine was 16.2% (95% CI 15.5–16.8) (21.9% women and 9.4% men).

**Table 1:** One-year prevalence of different headache types^1^ in HUNT4

Types of headache	Women (*n* = 23,635) %, (95% CI)	Men (*n* = 17,825) %, (95% CI)	Overall, crude^2^ (*n* = 41,460) %, (95% CI)	Adjusted for age and sex^3^%, (95% CI)
Headache sufferer	45.0 (44.0 to 46.0)	26.9 (25.9 to 27.9)	37.2 (36.5 to 37.9)	37.3 (37.0 to 37.6)
Migraine	15.3 (14.8 to 15.7)	5.7 (5.3 to 6. 0)	11.1 (10.8 to 11.4)	11.2 (10.9 to 11.5)
Episodic migraine < 15 days/month	14.0 (13.6 to 14.5)	5.3 (5.0 to 5.6)	10.3 (10.0 to 10.6)	10.3 (10.0 to 10.6)
Chronic migraine (≥15 days/month)	1.1 (1.0 to 1.3)	0.3 (0.2 to 0.4)	0.8 (0.7 to 0.9)	0.8 (0.7 to 0.9)
Migraine with aura	8.2 (7.8 to 8.5)	2.8 (2.5 to 3.0)	5.8 (5.6 to 6.1)	5.9 (5.7 to 6.2)
Migraine without aura	7.1 (6.8 to 7.4)	2.9 (2.7 to 3.2)	5.3 (5.1 to 5.5)	5.3 (5.1 to 5.5)
Probable migraine	5.8 (5.5 to 6.1)	3.5 (3.2 to 3.7)	4.8 (4.6 to 5.0)	4.8 (4.6 to 5.0)
TTH^4^% (95% CI)	23.1 (22.6 to 23.7)	17.4 (16.8 to 17.9)	20.7 (20.3 to 21.1)	20.7 (20.3 to 21.1)
Infrequent episodic TTH < 1 days/month	5.7 (5.3 to 6.0)	5.3 (4.9 to 5.6)	5.5 (5.3 to 5.7)	5.5 (5.3 to 5.7)
Episodic TTH 1–14 days/month	16.2 (15.7 to 16.7)	11.3 (10.8 to 11.7)	14.1 (13.7 to 14.4)	14.1 (13.7 to 14.4)
Chronic TTH (> 14 days/month	1.1 (1.0 to 1.3)	0.8 (0.7 to 0.9)	1.0 (0.9 to 1.1)	1.0 (0.9 to 1.1)
Medication overuse headache	0.3 (0.3 to 0.4)	0.2 (0.1 to 0.2)	0.3 (0.2 to 0.3)	0.3 (0.2 to 0.3)
Unclassified headaches^5^	0.8 (0.7 to 0.9)	0.3 (0.2 to 0.4)	0.6 (0.5 to 0.7)	0.6 (0.5 to 0.7)

1 Mutually exclusive headache diagnoses: The diagnosis of definite migraine excluded the diagnosis of TTH, and TTH excluded probable migraine. The diagnosis of MOH excluded the diagnoses of chronic TTH, chronic migraine and chronic unclassified headache.

2 Crude: Not age- and sex-adjusted

3 Adjusted for age- and sex using all invited participants in HUNT3 as the standard population

4 TTH = tension-type headache

5 Unclassified headache: Not fulfilling criteria of migraine, probable migraine or THH

### Time trends from HUNT3 and HUNT4

As demonstrated in Table 2, a higher proportion of participants in HUNT4 reported to suffer from headache (37.3% vs. 36.2%, *p* < 0.001) and TTH (20.7% vs. 15.9%, p < 0.001). In contrast, the 1-year prevalence of migraine (11.1% vs. 12.0%, p < 0.001) and MOH (0.3% vs. 1.0%, p < 0.001) were lower in HUNT4 than in HUNT3.

**Table 2:** Age-and sex adjusted 1-year prevalence of different headache types^1^ in HUNT3 and HUNT4

Headache type	HUNT3 (*n* = 39,697) %, (95% CI)	HUNT4 (n = 41,460) %, (95% CI)	*P* values
Headache sufferer	36.2 (35.9 to 36.5)	37.3 (37.0 to 37.6)	0.002
Migraine	12.0 (11.5 to 12.5)	11.2 (10.9 to 11.5)	< 0.001
Episodic migraine < 15 days/month	11.7 (11.4 to 12.0)	10.3 (10.0 to 10.6)	< 0.001
Chronic migraine (≥15 days/month)	0.3 (0.2 to 0.4)	0.8 (0.7 to 0.9)	0.007
Migraine with aura	5.9 (5.7 to 6.2)	5.9 (5.7 to 6.2)	0.11
Migraine without aura	6.0 (5.7 to 6.2)	5.3 (5.1 to 5.5)	< 0.001
Probable migraine	7.4 (7.1 to 7.7)	4.8 (4.6 to 5.0)	< 0.001
TTH^2^	15.9 (15.6 to 16.3)	20.7 (20.3 to 21.1)	< 0.001
Infrequent episodic TTH < 1 days/month	4.5 (4.3 to 4.7)	5.5 (5.3 to 5.7)	< 0.001
Episodic TTH 1–14 days/month	10.8 (10.5 to 11.1)	14.1 (13.7 to 14.4)	< 0.001
Chronic TTH (> 14 days/month)	0.6 (0.5 to 0.7)	1.0 (0.9 to 1.1)	< 0.001
Medication overuse headache (MOH)^1^	1.0 (0.9 to 1.1)	0.3 (0.2 to 0.3)	< 0.001
Unclassified headaches^3^	1.1 (1.0 to 1.2)	0.6 (0.5 to 0.7)	< 0.001

1 Mutually exclusive headache diagnoses: The diagnosis of definite migraine excluded the diagnosis of TTH, and TTH excluded probable migraine. The diagnosis of MOH excluded the diagnoses of chronic TTH, chronic migraine and chronic unclassified headache.

2 *TTH* tension-type headache

3 Unclassified headache: Not fulfilling criteria of migraine, probable migraine or THH

### Long term prognosis in a 22-years’ follow-up

**Fig. 2:**
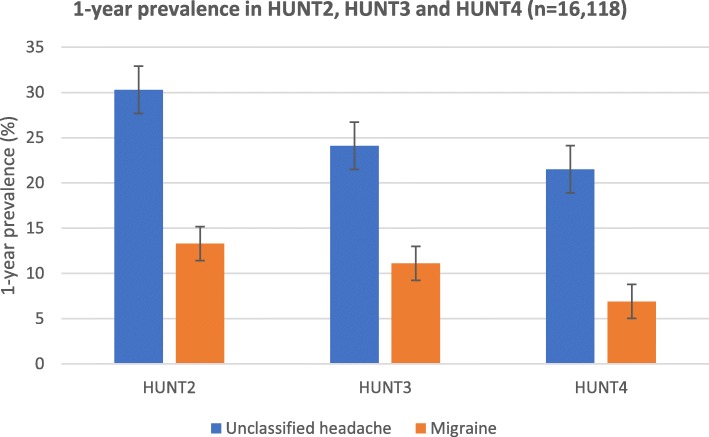
Development of migraine and headache prevalence among those who participated in HUNT2, HUNT3, and HUNT4 (n = 16,118).

Among individuals who participated in all three surveys (*n* = 16,118), the mean age increased from 44.2 years (95% CI 44.1–44.4) at baseline in HUNT2 to 65.9 years (95% CI 65.8–66.1) at the end of follow-up in HUNT4. As demonstrated by Table 3, all headache types markedly decreased during whole the follow-up period. For example, 1-year prevalence of migraine decreased from 13.3% at baseline in HUNT2 to 6.9% at end of follow-up in HUNT4 (Fig. 2).

**Table 3:** One-year prevalence of headache^1^ in HUNT2, HUNT3 and HUNT4 among individuals who participated in all three surveys (n = 16,118)

Headache type	HUNT2%, (95% CI)	HUNT3%, (95% CI)	HUNT4%, (95% CI)
Headache sufferer	44.4 (43.7 to 45.2)	36.1 (35.5 to 36.7)	28.8 (28.2 to 29.4)
Migraine	13.3 (12.7 to 13.7)	11.1 (10.6 to 11.6)	6.9 (6.5 to 7.3)
Episodic migraine < 15 days/month	12.7 (12.2 to 13.2)	10.5 (10.1 to 11.0)	6.4 (6.0 to 6.8)
Chronic migraine (≥15 days/month)	0.6 (0.5 to 0.7)	0.6 (0.5 to 0.7)	0.5 (0.4 to 0.6)
Migraine with aura	4.8 (4.5 to 5.1)	5.6 (5.2 to 5.9)	3.6 (3.3 to 3.9)
Migraine without aura	8.5 (8.0 to 8.9)	5.5 (5.2 to 5.9)	3.3 (3.0 to 3.5)
Medication overuse headache	0.8 (0.7 to 1.0)	0.9 (0.8 to 1.1)	0.4 (0.3 to 0.5)
Unclassified headaches^2^	30.3 (29.5 to 31.1)	24.1 (23.5 to 24.7)	21.5 (21.0 to 22.0)

1 Mutually exclusive headache diagnoses: The diagnosis of migraine excluded the diagnosis of TTH, and TTH excluded probable migraine. The diagnosis of MOH excluded the diagnoses of chronic migraine and chronic unclassified headache

2 Unclassified headache: Not fulfilling criteria of migraine

Among the individuals with migraine at baseline HUNT2 (*n* = 2136), at 21.7 years follow-up in HUNT4 a total of 40.6% were without headache, 33.2% had probable (*n* = 168) or definite (*n* = 541) migraine, 24.1% TTH, 1% MOH and 1% unclassified headache. Furthermore, among 4945 with non-migrainous headache in HUNT2, 58.8% were without headache, 28.5% had TTH, 11.6 had probable (*n* = 228) or definite (*n* = 345) migraine, 0.6% had MOH and 0.5% unclassified headache in HUNT4.

A total of 8956 individuals were without headache in HUNT2. At follow-up in HUNT4, 85.7% still were without headache, 10.3% had TTH, 3.8% had probable (*n* = 147) or definite (*n* = 195) migraine, 0.1% had MOH and 0.1% unclassified headache.

### Risk factors in a 22-years’ follow-up

Participants in the age group 20–39 years at baseline had nearly three times increased risk (RR = 2.8, 95% CI 2.5–3.1) of reporting headache in all three surveys compared to persons aged 50 years or more (Table 4). Female sex, occurrence of chronic musculoskeletal complaints and high score of depression or anxiety at baseline doubled the risk of having headache in all three surveys (Table 4). Current and past smoking slightly increased the risk (RR1.2, 95% CI 1.1–1,2) of having stable headache, whereas as lower risk was found for elevated systolic blood pressure (BP) (RR 0.6, 95% CI 0.5–0.8), for physical inactivity (RR 0.8, 95% CI 0.7–0.9), and among those who drank alcohol at least 5 times during the last month (RR 0.7, 95% CI 0.7–0.8). Overall, compared to the group with stable headache, the RRs were slightly lower for those with new-onset headache, and even lower for those with previous headache (Table 4).

**Table 4:** Poisson regression was used to evaluate baseline variables in HUNT2 and risk ratios (RRs) of headache status 22 years later in HUNT4 in persons who participated in all three surveys (n = 16,118). Precision of the estimates was assessed by 95% confidence interval (CIs). Reference group in all three headache status groups consisted of participants without headache in all three surveys (*n* = 6775)

	Previous headache^1^ (*n* = 4700)		New-onset headache^2^ (*n* = 1902)		Stable headache^3^ (*n* = 2741)	
Demographic variables^**4**^	RR	95% CI	RR	95% CI	RR	95% CI
Age 20–39 years	1.4	1.3–1.5	2.1	1.9–2.3	2.8	2.5–3.1
40–49 years	1.2	1.1–1.3	1.6	1.5–1.8	1.9	1.7–2.1
50 years or more (reference)						
Gender (women vs. men)	1.5	1.4–1.5	1.7	1.6–1.9	2.3	2.1–2.5
Education (low vs. high)	1.0	1.0–1.1	1.2	1.0–1.4	1.1	1.0–1.2
**Headache status at baseline**^**5**^						
Migraine (yes vs. no)	2.5	2.5–2.6	3.8	3.5–4.1	3.7	3.6–3.9
Migraine with aura (yes vs. no)	2.3	2.2–2.4	3.7	3.3–4.1	2.8	2.6–2.9
Migraine without aura (yes vs. no)	2.5	2.4–2.5	3.7	3.3–4.0	3.0	2.8–3.1
Non-migrainous headache (yes vs. no)	4.6	4.4–4.8	4.6	4.3–4.9	5.6	5.3–5.9
Chronic headache without MOH^3^ (yes vs. no)	2.1	1.9–2.3	2.1	1.6–2.8	2.6	2.4–3.0
MOH^6^ (yes vs. no)	2.3	2.1–2.5	4.1	2.5–6.8	3.3	2.9–2.7
**Self-reported complaints**^**2**^						
Chronic MSCs (yes vs. no)	1.4	1.4–1.5	1.6	1.5–1.7	2.1	2.0–2.2
HADS A score (> 11 vs. < 7)^7^	1.5	1.4–1.7	1.8	1.5–2.1	2.1	1.9–2.3
HADS D score (> 11 vs. < 7)^7^	1.5	1.3–1.7	1.4	1.1–1.9	2.1	1.9–2.5
**Measured variables**^**5**^						
BMI^8^ (> 30 vs. < 25)	1.1	1.0–1.1	1.0	0.9–1.1	1.1	1.0–1.2
Systolic BP (> 160 mmHg vs. < 140 mmHg)	1.0	1.0–1.2	0.7	0.5–0.8	0.6	0.5–0.8
**Lifestyle factors**^**5**^						
Smoking (daily or past vs. never)	1.1	1.0–1.1	1.1	1.1–1.2	1.2	1.1–1.2
Days using alcohol per month (≥5 vs. 0)	0.8	0.7–0.9	1.0	0.8–1.1	0.7	0.7–0.8
Inactive vs. high physical activity^9^	0.9	0.8–1.0	0.9	0.8–1.1	0.8	0.7–0.9

1. Headache in HUNT2 and/or HUNT3, but without headache in HUNT4. 2. Without headache in HUNT2 and/or HUNT3 but reported headache in HUNT4. 3. Headache in HUNT2, HUNT3 and HUNT4. 4. Adjusted for age, gender and education level 5. Adjusted for age, gender, education and measured variable^;^ 6. Medication overuse headache; 7. Hospital Anxiety and depression scale; 8. Body mass index; 9. Reference High (≥3 h hard activity per week) vs. physical inactive (none or less than 1 h of light activity per week)

In supplementary analyses evaluating the influence of age, highest RRs for having stable headache at follow-up were found for baseline factors like headache subtypes and self-reported complaints among those aged 50 years and older in HUNT2, and lowest RR among those aged 20–39 years in HUNT2. E.g., individuals aged 50 years or more with chronic musculoskeletal complaints at baseline had an increased RR of 3.1 (95% CI 2.5–3.8) of reporting headache in all three surveys, whereas the corresponding RR was 1.8 (95% CI 1.6–1.9) for those aged 20–40 years in HUNT2. Similarly, among persons aged 50 years or more with migraine or MOH at baseline, RRs for having stable headache were respectively 7.3 (95% CI 6.-8.2) and 6.2 (95% CI 5.2–7.3), whereas the corresponding RRs were 2.8 (95% CI 2.6–2.9) and 2.0 (95% CI 1.8–2.3) among those aged 20–39 years. Furthermore, regarding elevated systolic BP, persons aged 50 years or more had the most prominent reduced risk (RR = 0.5, 95% CI 0.3–0.8) of having stable headache.

## Discussion

The main findings by using population-based data from two surveys were that the 1-year prevalence of migraine and MOH was lower in HUNT4 than in HUNT3, whereas prevalence of headache and TTH increased. In a 22-years’ follow-up among participants in three surveys, young age, female sex, and occurrence of musculoskeletal complaints and high score of anxiety and/or depression at baseline were all associated with substantially increased risk of reporting headache in all three surveys.

### Time trends

The prevalence of TTH increased from 15.9% to 20.7% from HUNT3 to HUNT4. In accordance, in a Danish study, using a neutral screening question, the prevalence of TTH increased from 79% to 87% in the 12-year follow-up [10]. Furthermore, increasing TTH prevalence from 5% in 2008 to 23% in 2013 was recently reported in two nationwide population-based studies performed in Turkey [23].

Interestingly, we found that the prevalence of migraine decreased from 12.0% to 11.1%. In contrast, some previous studies have demonstrated an increased migraine prevalence, in Denmark from 11% to 15% from 1989 to 2001 [10], in Spain from 6.5% to 9.7% between 2003 and 2012 [5], and in Norway from 12.1% to 13.2% from 1995 to 1997 to 2006–2008 based on criteria that included self-reported migraine [6]. However, by comparing HUNT data in the period from 1995 to 2019, the 1-year prevalence of migraine of 12.1, 12.0 and 11.1, respectively, shows that the migraine prevalence in Nord-Trøndelag county is not increasing; and rather, there is a slightly decrease. Hopefully, this could reflect more healthy lifestyles or improved treatment of headache. A stable prevalence of definite migraine (16.4% in 2008 and 16.7% in 2013) was also found in the Turkish study [23].

We demonstrated for the first time a recent decrease in the prevalence of MOH from 1.0% in HUNT3 to 0.3% in HUNT4. It should be highlighted that the questionnaire-based diagnosis of MOH in HUNT3 and HUNT4 was based on identical phrasing of questions regarding headache and use of analgesics. The reason for the marked decrease in MOH prevalence is unclear. In the present study we have no documentation that this is explained by better health care. On the other hand, we may hope that it reflects increased use of preventive medication and more focus on restricted use of pain killers in the primary care. In both HUNT3 and HUNT4, we could have underestimated the prevalence of MOH. Firstly, the validity of the headache screening question was not optimal, and several individuals with MOH may have been missed because they answered “no” to the screening question. Secondly, our questionnaire-based diagnosis of MOH (use of pain killers at least 4 days/week) implied use of about ≥17 days/month rather than the ≥15/days/month limit according to the ICHD 3 criteria [17]. Thirdly, we could have underestimated the prevalence of MOH because the diagnosis was based on over-the-counter (OCT) medication only and not taking triptan or opioid overuse into consideration. On the other hand, in HUNT3 we collected data on both OCTs and prescribed medicines, the latter by the Norwegian Prescription Database (NPD). With this method, we captured 2/3 of patients with MOH trough the information about OCTs, and only 1/3 were identified through the NPD, overusing triptans and/or opioids [24]. Similarly, the vast majority with MOH overused over-the-counter analgesics in a recent large population-based study from Denmark, whereas only 12% overused triptans and 23% opioids [25]. Interestingly, the prevalence of MOH was munch higher in the Danish study (2.0%) than in the present study (0.4%). The reason for the difference is unclear but may in part be related to above mentioned underestimation of the diagnosis MOH in the present study. More speculatively, the difference could also in part be related to differences in prescription of analgesics and/or genetic, psychosocial, behavioral factors and ethnicity. Among participants in the HUNT studies, there is a relatively low proportion of immigrants [16], whereas in the Danish study the highest MOH prevalence was found among non-Danish, non-western participants [25].

### Prognosis

During the 22-year follow-up, 53.4% of individuals who suffered from headache in HUNT2 were without headache in HUNT4, whereas 12.5% fulfilled the migraine criteria, 27.2% TTH, 0.7% MOH and 6.2% other types of headache. In comparison, during 40-years follow-up of school children with severe migraine in Sweden, less than half did not report having migraine at follow-up [13]. In a 12-year follow-up Denmark, remission occurred in 45% of persons with TTH and 42% with migraine, whereas 16% with TTH and 6% with migraine had headache more than 14 days/month. In a 30 years prospective study from Switzerland, the vast majority of persons had transient migraine and TTH [12]. Furthermore, 16% of participants with migraine and 29% with TTH was headache-free in the Turkish 5-year follow-up study [23].

### Risk factors

In the present study young age and female gender were important factors for poor prognosis defined as reporting headache in all three surveys. In accordance, such factors are also reported as poor prognostic factors in the follow-up study from Denmark [11]. We identified two different patterns regarding age. On the one hand, because prevalence of headache decreases with increasing age (Fig. 2), it is reasonable that participants in the lowest age group are more likely to be a headache sufferer at follow-up compared to the oldest group. On the other hand, the impact of baseline factors on RR of having stable headache at follow-up was most prominent for individuals aged 50 years or more, and least prominent for those aged 20–39 years. For example, individuals having musculoskeletal complains or anxiety/depression after the age of 50, were more likely to suffer from headache than younger people with the same risk factors.

In this study we also found that occurrence of chronic musculoskeletal complaints and high score of depression or anxiety at baseline doubled the risk of having headache in all three surveys. This is in accordance with our previous report of a bidirectional association between headache and chronic musculoskeletal complaints [26], and a cross-sectional association between elevated HADS score and headache [27]. Furthermore, chronic musculoskeletal complaints and high score of depression or anxiety at baseline were also important risk factors for developing migraine and MOH in an 11-year follow-up [24, 28]. Co-occurrence of headache with other complaints like chronic musculoskeletal complaints, depression and anxiety probably reflect a complex and bidirectional causal relationship, and possibly also common underlying factors. It may also indicate a potential for improving the long-term prognosis of headache if co-morbid conditions are treated.

We found a slightly increased risk of stable headache in current and past smokers. The mechanism behind this finding is unclear and likely complex. However, it should be highlighted that a Mendelian randomization analysis based on the HUNT study did not find evidence of a strong relationship between smoking and headache [29]. Thus, it seems premature to conclude from the present study that smoking can cause persistent headache.

Finally, we found that high systolic BP and frequent use of alcohol were associated with lower risk of having stable headache. This is in accordance with our previously results that high BP lower the risk of developing headache [30], and that alcohol use is associated with lower prevalence of migraine [31]. The lower risk of stable headache among those who consumed alcohol as compared to abstainers, is likely to reflect the fact that alcohol is a common trigger of headache (reversed causality) [28]. Conceivably, individuals who at baseline in HUNT2 already had experienced that alcohol causes headache may tend to avoid it [28]. In previous prospective HUNT studies, we have found that physical activity gave lower risk of headache and migraine compared to physical inactivity [25, 32]. Contrary to these results, in the present study physical inactivity was associated with lower risk of having stable headache. We have at present no good reasonable explanation for this discrepancy, but it might be related to a general advice, giving during the last years, that headache sufferers will benefit from physical activity.

### Strengths and limitations of the study

- The major strengths of this study are the longitudinal population-based cohort design with many participants, a wide age range, and the use of validated diagnoses of headache [14, 19, 20]. Direct comparison of 1-year prevalence between HUNT3 and HUNT4 was meaningful because the questionnaire-based diagnosis of migraine, TTH and MOH were based on identical phrasing of the headache questions, and because we could adjust for age and sex in both. In the longitudinal analysis of risk factors at baseline, we adjusted for the same confounding factors in all analyses, making the estimated RRs for the different health related factors comparable.

Several study limitations should also be considered. Firstly, generalization of the results to the entire population must be made with some caution, since the participation rate was 42–55% in the three surveys, and only 17% of the invited population in HUNT2 participated in all three surveys, and we cannot be certain that loss to follow-up was random. Secondly, in the longitudinal study subjects were subclassified according to the answer to the screening question “Have you suffered from headache during the last 12 months?”. It should be highlighted that those without headache also included those who experienced headache without defining themselves as being headache sufferers. In fact, 74% of participants in the validation study of HUNT3 reported having had headache during the last year, whereas only 31% stated that they had suffered from headache during the same period [33].

## Conclusion

In a 11-years’ follow-up, the1-year prevalence of migraine and MOH decreased, whereas prevalence of headache and TTH increased. In a 22-years’ follow-up, young age, female sex, and occurrence of musculoskeletal complaints and high score of anxiety and/or depression at baseline increased the risk of reporting headache at end of follow-up, whereas elevated systolic blood pressure at baseline lower risk of headache at follow-up.

## Data Availability

Part of the dataset supporting the conclusions of this article is available on request to the corresponding author. Some of the data are the property of HUNT research centre and can only be accessed through direct contact with. the research centre.

## Abbreviations

HUNT: The Nord-Trøndelag Health Survey
Q1: First questionnaire
Q2: Second questionnaire
ICHD-3: The International Classification of Headache Disorders 3rd edition
TTH: Tension-type headache
MOH: Medication-overuse headache
CI: Confidence interval
MA: Migraine with aura
MO: Migraine without aura
